# What will robots be like in the future?

**DOI:** 10.1093/nsr/nwz069

**Published:** 2019-06-07

**Authors:** Yanfeng Lu, Weijie Zhao

**Affiliations:** State Key Laboratory of Management and Control for Complex Systems, Institute of Automation, Chinese Academy of Sciences

*Robots are changing our lives: sweeping robots patrol our living rooms; interactive robots accompany our children; industrial robots assemble vehicles; rescue robots search and save lives in catastrophes; medical robots perform surgeries in hospitals. To better understand robots' challenges and impact, National Science Review (NSR) interviewed Professor Toshio Fukuda, who is one of the world’s most representative robotics experts and has developed a number of bionic robots and micro/nano-robots.*

*Fukuda has been a full-time professor at Beijing Institute of Technology (BIT) since 2013. Before that, he served as a professor at Nagoya University in Japan for more than 20 years. Fukuda is now a foreign member of the Chinese Academy of Sciences and has cultivated many robotic researchers for China. He has been elected as the 2020 president of the Institute of Electrical and Electronic Engineers (IEEE), which means that he will play a central leading role in the world's largest technical professional organization in the coming years.*

## WHAT IS A ROBOT?

**NSR:** What do you think is the definition of a robot? Why do we consider unmanned aerial vehicles (UAVs) as robots but do not consider common airplanes as robots?

**Fukuda:** If a flying vehicle is autonomous in some degree, we can consider it as a flying robot. By my definition, robot is such a kind of machine that has sensors, actuators, as well as inside or outside central processing units (CPUs).

The extent of automation is various. Industrial robots, which were defined by the International Standard Organization (ISO) as programmable robots with three or more degrees of freedom, can only do what they are programmed to but cannot make decisions by themselves. Many other robots, such as a number of medical robots are also strictly programmed. We do not allow the medical robots themselves to decide what to do in our bodies. They should follow the indication of doctors.

However, the intelligent robots are different. They can sense the environment and use their own CPUs to make decisions according to the environmental changes. There are two major types of intelligent robots, the teleoperated robots and the autonomous robots. The teleoperated robots interact with human closely and make decisions with human's help. The famous cartoon robot ‘Gundam’ is a teleoperated robot. On the other hand, the autonomous robots do not need human to make decisions. There are also half-teleoperated half-autonomous robots. One example is the Mars rover. When it lands on Mars, it takes photos and decides which way to go under the commands of the scientists on the Earth. However, on its way, it has to sense the obstacles such as rocks and decides how to navigate around the obstacles by itself. Some medical micro-robots are also intelligent. We can inject them into patients’ bodies; then they can navigate to the diseased region or other target organs autonomously and perform microsurgeries collaborating with doctors.

**Figure:**
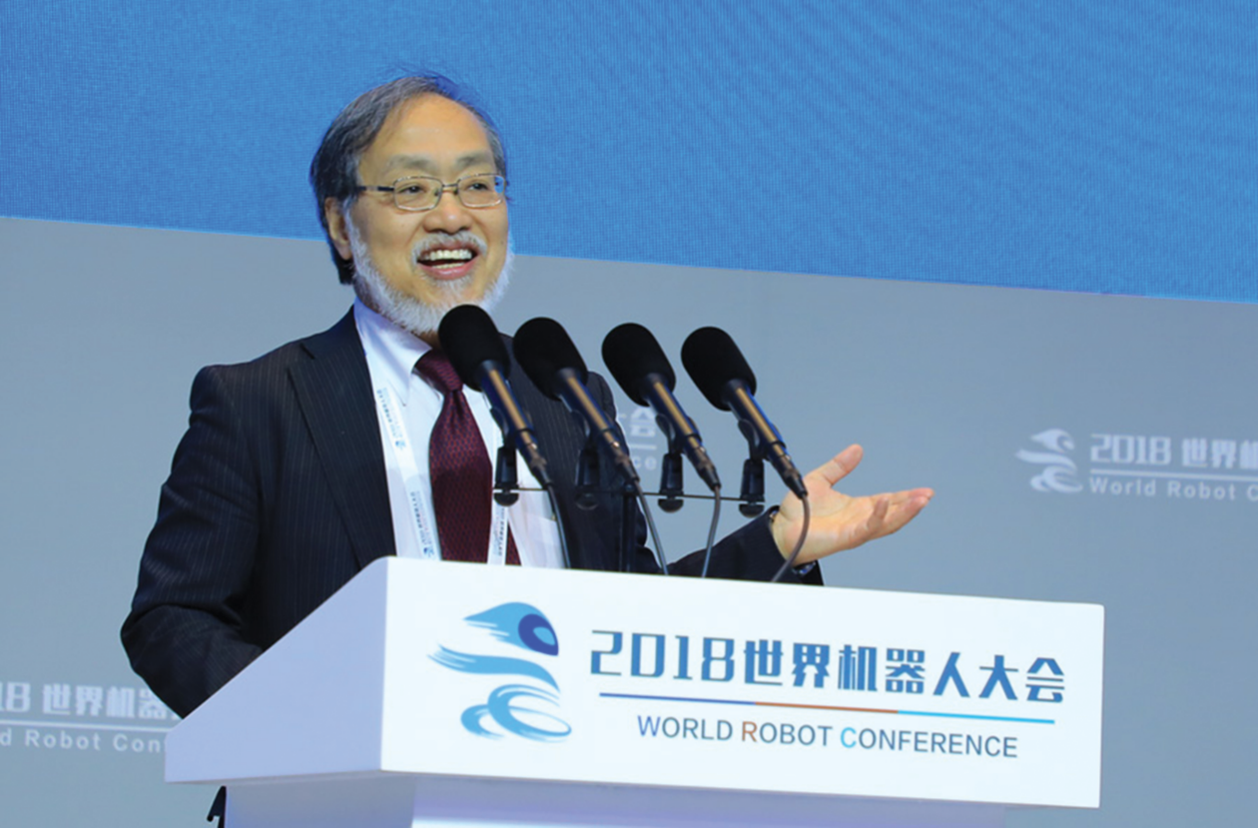
Professor Fukuda on the 2018 World Robot Conference, Beijing *(Courtesy of Toshio Fukuda)*.

## GRAND CHALLENGES OF ROBOTICS

**NSR:** What are the current grand challenges of robotics?

**Fukuda:** The grand challenges of robotics in my mind go with the megatrends of human society. I do not talk about the challenges of 5 or 10 years, but the challenges of the next generations 50 or 100 years ahead. Robotics should help human cope with the vital problems we are facing.

Robotics should help human cope with the vital problems we are facing.

—Toshio Fukuda

The first megatrend is the aging society. China, Japan and many other countries are currently facing an aging society with a demographic inverted pyramid. There will be more and more senior people in our society, and how can we cope with it with robotics? Medical robots can help to analyse, diagnose and treat diseases. Better industrial robots can make it easier to work in factories so that senior people can work until a higher age. Escort robots can help seniors increase their quality of life. There are many things robotics can do and should do.

The second megatrend is global warming. It is very likely that the Earth's climate will change significantly in the coming decades. Many lands may become desert and untillable. There will be difficulties for some areas to obtain water and food. Robotics can make agriculture more autonomous and effective. A water supplemental and recycling system will also help to reduce agricultural water consumption. This kind of new agriculture system has already been tested in several countries including China.

The third vital problem we are facing is the energy problem. What should we do if fossil energy is exhausted? Robotics can help us to harvest energy from everywhere. We can place a small turbine in the toilet to harvest energy from the flush water, or design a device to harvest energy from the opening door, or use the wearable devices to harvest energy from our own body movement. All kinds of motion can be utilized.

Another megatrend is artificial intelligence (AI). It is said that there will be a singularity in 2045, when intelligent robots will become smarter than humans.

I talked about four megatrends here, but there are actually more. We should prepare for these problems with the help of robotics in order to avoid possible catastrophes.

**NSR:** When the robots become smarter than humans, there will be ethical problems?

**Fukuda:** That's right. My IEEE friends and I are working on the ethic design of robots. We should ensure by technology that robots, such as self-driving vehicles, would not harm humans. Many technological companies, such as Baidu which is cooperating with my group in BIT, are working on these issues.

## MICRO-REPAIRMEN IN OUR BODIES

**NSR:** Would you please give some examples of medical micro/nano-robots?

**Fukuda:** BIT professor Shuxiang Guo developed an assistant system for minimally invasive vascular surgery when he was my student in Japan. With this system, doctors can make an incision somewhere on the patient's body and insert a one-millimeter-diameter catheter as well as a guide wire into the blood vessel. With the help of multiple sensors, the catheter and guide wire can be navigated along the blood vessel towards the distant diseased organ, such as the brain. Then, doctors can perform microsurgeries such as dredging the chocked up blood vessels or placing vascular stents. This system has already been used in many hospitals in and out of China, and researchers are still looking for further advancements.

**Figure:**
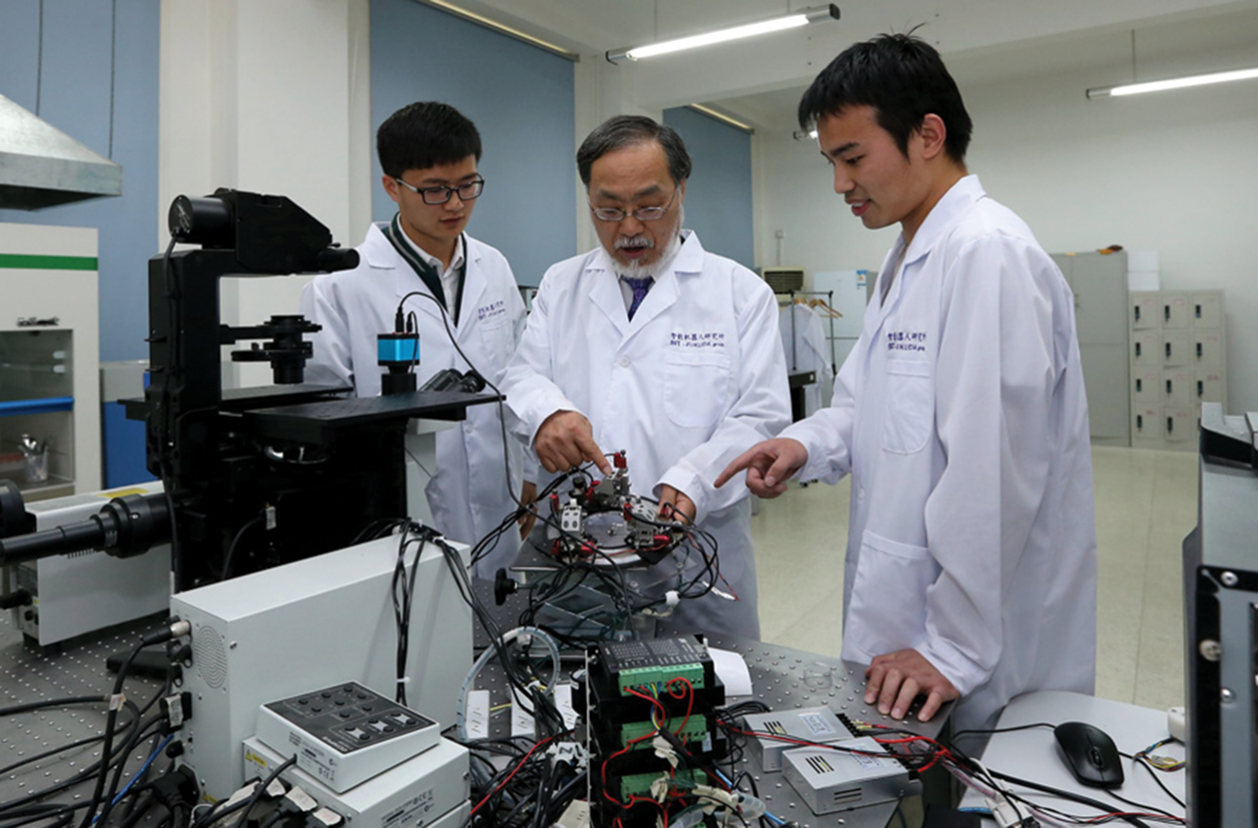
Professor Fukuda with students in his BIT lab *(Courtesy of Toshio Fukuda)*.

Another example is our artificial micro-vascular. My group has successfully assembled artificial micro-vascular as long as 200 micrometers in the laboratory. I hope that in the future, when human tissues went wrong, we can use this kind of cell-level micro-technology to repair the broken tissues *in situ*. But of course, we should go step by step, test the technologies in animals before using them in the hospitals.

**NSR:** Why is it difficult to make micro-robots?

**Fukuda:** In the macro world, gravity is the leading force. But when the size becomes smaller and smaller, gravity becomes unimportant and the impact of surface forces become significant. So micro-engineering is different from macro-engineering. It is not easy to make micro-robots, and especially difficult to make durable micro-robots.

**NSR:** How is micro/nano-robotics developing in China?

**Fukuda:** I brought this research field into China. Now, there are four or five Chinese groups doing very well, most of which are led by my Chinese students. I am very glad to see my students spreading across China, in Shenyang, Wuhan, Suzhou, Shanghai, Shenzhen and Hong Kong.

## CHINESE STUDENTS AND CHINA’S ROBOTIC INDUSTRY

**NSR:** Why did you join a Chinese university in your 60s?

**Fukuda:** I have many Chinese students and they are very energetic and enthusiastic. In Japan, the society is more mature and the best students always join big companies and live a rich and stable life. But Chinese students are different. Many of them are very energetic and ambitious. They work very hard and would like to create their own companies with the most advanced technologies. That is why I like China.

**NSR:** What suggestions would you give to the young scientists?

**Fukuda:** My job as a professor is to give dreams to the young generation. So my suggestion is to have a good dream and keep going.

Everybody has his or her own dream. I know someone who dreams to develop an air-exchange system. And he is now trying hard to find materials that could absorb moisture from the air. I once dreamed about improving people's sleep. So in Nagoya University, we analysed the rhythm of human and developed a biocompatible and biodegradable micro-capsule, which is made of liposomes containing proteins that can control sleep conditions. We hope that it will turn into a useable medicine one day.

So it is important to have a dream and work hard to realize it with science and technology.

**NSR:** Japan's robotic industry is one of the world’s best. How could China catch up?

**Fukuda:** I discussed this issue with one of my best Chinese friends about 15 years ago. At that time, I said that there should be four steps. Step 1, you should observe and study the state-of-the-art foreign robots carefully. Step 2, you should digest the foreign technology and make a robot as well as the existing best ones. Step 3, you should improve it. Step 4, you can create a completely new and better robot.

Now, China's robotic industry has developed a lot and is ready to break into step 4. A number of Chinese robot companies, such as SIASUN Robot and Automation Corporation, are making very nice robots. SIASUN’s automatic guided vehicles (AGVs) are the best in China.

However, there may be something still missing. One of the limitations is that China has not a strong component industry. China need to import many basic components from Japan and other countries. The Chinese government exposed the China Manufacturing 2025 plan to solve this problem. But it may still need years to decode and catch up with the foreign technologies.

**NSR:** You helped to organize the first Beijing World Robot Conference (WRC) in 2015. How would you evaluate this conference?

**Fukuda:** There are several similar conferences in other countries, and China also wanted to organize its own. I was invited as the Chair of the Advisory Committee of this new conference and helped to contact the robotic specialists, many of whom are my friends, such as MIT professor Rodney Brooks, who is one of the founders of the company iRobot, which is famous for its robotic vacuum cleaner Roomba. At last, we had more than 50 senior scientists in the first WRC.

WRC has been very successful in the past years. It makes it possible for Chinese people to know what is going on in the world within one week. There are forums, exhibitions and contests in the conference, both the scientists and the public can enjoy it.

## AN IEEE UNIVERSITY OPENS FOR ALL

**NSR:** You have been selected as the 2020 president of IEEE. What are your major missions in this position?

**Fukuda:** IEEE is a non-profit organization with 430 000 members and 46 technical societies and councils, covering diverse fields including computer sciences, robotics, electronics, medical engineering and more. Our aim is to advance technology for humanity. As the president, I wish to make all IEEE members connected as families.

Particularly, one of my promises during the election was to build an ‘IEEE University’. It will not be a real university, but virtual, consisting of a massive open online courses (MOOC) system. Many of our societies already have their own online courses and I want to assemble them into a more efficient and more user-friendly system. All of our courses will be open and free to anybody anywhere in the world. You do not need to pay anything unless you want a course certification for job or university applications. We will start to prepare for it in 2019, and build the system in 2020. Once the frame is built, this online university will naturally grow day by day.

It [the ‘IEEE University’] will not be a real university, but virtual, consisting of a massive open online courses (MOOC) system.

—Toshio Fukuda

**NSR:** What are your personal plans in the coming five years?

**Fukuda:** I have two major aims in the coming years. First is to keep my research group at BIT as the best micro/nano-robotics group in the world. And the second is to contribute more to IEEE.

Both of the aims require a lot of communications with people. I need to communicate with my group members in Beijing. I also need to communicate with the staffs of IEEE in the US. I should listen carefully to their voices and make decisions. Fortunately, the communication technologies are highly developed now, so that I would be able to handle these jobs.

## Notes

Yanfeng Lu is an associate professor at the State Key Laboratory of Management and Control for Complex Systems, Institute of Automation, Chinese Academy of Sciences; Weijie Zhao is an NSR news editor.

